# Recent Advances in SERS-Based Detection of Organophosphorus Pesticides in Food: A Critical and Comprehensive Review

**DOI:** 10.3390/foods14213683

**Published:** 2025-10-29

**Authors:** Kaiyi Zheng, Xianwen Shang, Zhou Qin, Yang Zhang, Jiyong Shi, Xiaobo Zou, Meng Zhang

**Affiliations:** 1 School of Food and Biological Engineering, Jiangsu University, Zhenjiang 212013, China; kaiyizheng@ujs.edu.cn (K.Z.); sxw18906320352@163.com (X.S.); 19599960979@163.com (Z.Q.); yangzhang1@ujs.edu.cn (Y.Z.); shi_jiyong@ujs.edu.cn (J.S.); 2 Department of Physics, East China University of Science and Technology, Shanghai 200237, China; mzhang@ecust.edu.cn

**Keywords:** Raman enhancement mechanisms, SERS substrate engineering, organophosphorus pesticides, food safety

## Abstract

Surface-enhanced Raman spectroscopy (SERS) has rapidly emerged as a powerful analytical technique for the sensitive and selective detection of organophosphorus pesticides (OPPs) in complex food matrices. This review summarizes recent advances in substrate engineering, emphasizing structure–performance relationships between nanomaterial design and molecular enhancement mechanisms. Functional groups such as P=O, P=S, and aromatic rings are highlighted as key determinants of Raman activity through combined chemical and electromagnetic effects. State-of-the-art substrates, including noble metals, carbon-based materials, bimetallic hybrids, MOF-derived systems, and emerging liquid metals, are critically evaluated with respect to sensitivity, stability, and applicability in typical matrices such as fruit and vegetable surfaces, juices, grains, and agricultural waters. Reported performance commonly achieves sub-μg L^−1^ to low μg L^−1^ detection limits in liquids and 10^−3^ to 10 μg cm^−2^ on surfaces, with reproducibility often in the 5–15% RSD range under optimized conditions. Persistent challenges are also emphasized, including substrate variability, quantitative accuracy under matrix interference, and limited portability for real-world applications. Structure–response correlation models and data-driven strategies are discussed as tools to improve substrate predictability. Although AI and machine learning show promise for automated spectral interpretation and high-throughput screening, current applications remain primarily proof-of-concept rather than routine workflows. Future priorities include standardized fabrication protocols, portable detection systems, and computation-guided multidimensional designs to accelerate translation from laboratory research to practical deployment in food safety and environmental surveillance.

## 1. Introduction

As global agricultural systems advance toward greater intelligence, efficiency, and sustainability, pesticides remain important chemical inputs for stabilizing yields, enhancing economic returns, and maintaining food security in the face of challenges such as population growth, climate variability, and pest outbreaks [1,2]. Among pesticide classes, organophosphorus pesticides (OPPs) have been widely applied because of their well-established insecticidal mechanisms, mature synthesis pathways, broad-spectrum efficacy, low cost, and relatively rapid degradation under certain conditions [3,4,5,6]. They are commonly used in the cultivation of grains, fruits, vegetables, and horticultural crops, although patterns of use and regulatory approval vary across regions and production systems [7,8]. At the same time, these benefits are accompanied by notable trade-offs, including the development of insecticide resistance, non-target effects on beneficial organisms, ecological risks to soil and aquatic systems, and residue concerns in food. Consequently, increasing regulatory scrutiny and stricter maximum residue limits (MRLs) highlight the need for sensitive and selective analytical methods to monitor OPPs and ensure food safety.

In recent years, the intensity of OPP application has increased in several agricultural systems, particularly in countries with high pesticide consumption such as China and India, as well as in certain regions of Latin America where regulatory enforcement and residue monitoring remain less stringent [9]. Under these conditions, residues of OPPs have been frequently reported in soil, water, and crops, while in contrast, surveys in the European Union and the United States often show lower detection frequencies due to stricter regulations and monitoring frameworks [10]. Once released into the environment, OPPs can migrate via surface runoff, soil leaching, and atmospheric dispersion, and in some cases enter food webs through trophic transfer, resulting in localized bioaccumulation and ecological risks. Epidemiological and toxicological studies further indicate that chronic exposure to low concentrations of OPPs may lead to adverse outcomes such as neurotoxicity, endocrine disruption, reproductive toxicity, immunosuppression, and potential carcinogenicity, depending on the specific compound, exposure dose, and duration [11]. Given the pervasive nature of OPP contamination and its serious implications, there is a pressing demand for sensitive, specific, rapid, and robust analytical techniques capable of detecting trace levels of OPPs across a wide range of complex sample matrices [12,13,14]. At present, conventional detection methodologies for OPPs primarily rely on chromatographic and immunoassay techniques, such as gas chromatography (GC), high-performance liquid chromatography (HPLC), gas chromatography–mass spectrometry (GC-MS), and enzyme-linked immunosorbent assay (ELISA) [15,16,17]. These techniques are well-validated for their high sensitivity, specificity, and accuracy under laboratory conditions and have formed the backbone of pesticide residue monitoring programs globally. However, these methods also exhibit several inherent limitations. They often require sophisticated and expensive instrumentation, involve complex and labor-intensive sample preparation processes, and necessitate highly trained personnel [18]. Moreover, the time-consuming nature of these procedures restricts their applicability for rapid, real-time monitoring, which is increasingly demanded in modern agricultural production, food supply chains, and environmental surveillance systems [19].

These challenges become particularly evident when analyzing samples with complex matrices, such as fruit and vegetable juices, soil extracts, and agricultural irrigation water, where matrix interferences and the coexistence of non-target analytes can severely compromise detection accuracy and reproducibility [20,21]. Furthermore, the growing emphasis on sustainable agriculture and environmental stewardship calls for analytical tools that are not only sensitive and specific but also adapted to the needs of different end-users. For regulatory and reference laboratories, high-throughput and cost-effective systems are essential for processing large numbers of samples with robust quality control, while at the farm gate or field level, portable and user-friendly devices are prioritized to enable rapid, on-site decision-making. Therefore, developing alternative detection platforms that overcome the limitations of conventional methods and address these diverse application scenarios represents a critical research frontier. Against this backdrop, SERS has garnered significant attention as a promising technique for the detection of trace-level contaminants [22,23], including pesticide residues. SERS combines the intrinsic advantages of Raman spectroscopy, including its unique molecular fingerprint identification capability, with the remarkable signal amplification produced by localized surface plasmon resonance (LSPR) from metal nanostructures, including silver (Ag) and gold (Au) [24]. Upon molecular adsorption onto these nanostructured surfaces, the Raman scattering intensity can be enhanced by factors of up to 10^6^ or more, theoretically enabling the detection of molecules at low concentration [25,26]. Compared to traditional chromatographic approaches, SERS offers distinct advantages, including minimal sample preparation, rapid analysis [27], non-destructive measurement, and effective operation in complex environments [28,29].

**Figure 1:**
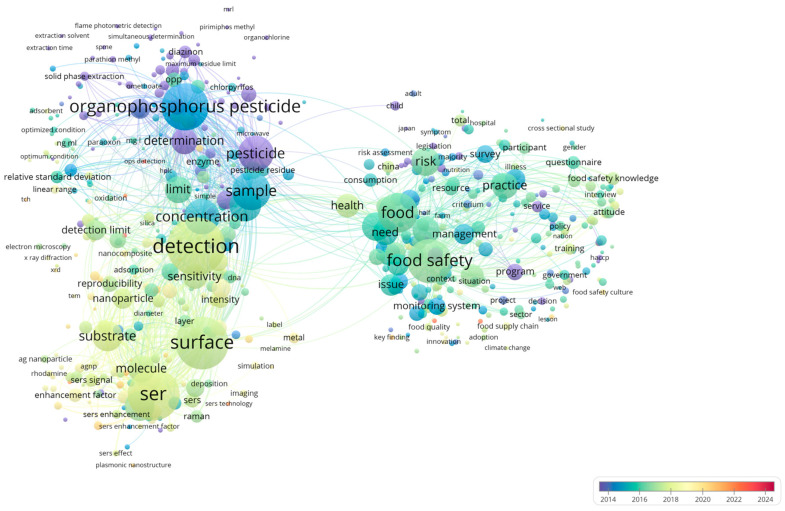
Keyword network diagram for SERS research.

Figure 1 highlights the key research areas of SERS in OPPs detection and food safety monitoring. SERS serves as a core technique in this field, with research primarily focusing on enhancing detection sensitivity, optimizing SERS substrates (e.g., nanoparticles and nanocomposites), and improving signal amplification through plasmonic nanostructures.

Notably, the high-resolution spectral information provided by SERS facilitates the discrimination of structurally similar OPPS, even those with only minor variations in functional groups. Extensive research has elucidated that specific functional groups within OPP molecules, such as P=O and P=S groups, aromatic rings, and halogen substituents, exhibit various interfacial interactions with noble metal nanoparticles, including coordination adsorption, electronic coupling, and π–π stacking [30,31]. These molecular interactions significantly influence the intensity and patterns of Raman signal enhancement. For example, P=O and P=S groups tend to form semi-covalent chemisorption bonds with metal surfaces [32], resulting in strong Chemical Enhancement (CE) effects, whereas aromatic rings often engage in weak physisorption [33,34,35], promoting prolonged surface residence times, enhanced molecular polarizability, and clearer skeletal vibrational peaks [36].

In addition to molecular-level interactions, the rational design and functional engineering of nanomaterial substrates are paramount to optimizing SERS performance. Substrate features such as abundant hot spots (regions of highly concentrated electromagnetic fields) [37], high surface uniformity, chemical stability, and strong analyte enrichment capabilities directly determine the sensitivity, reproducibility, and robustness of SERS measurements [38]. Recent advances in nanofabrication technologies, including the development of hybrid nanostructures, hierarchical assemblies, and surface modification strategies, have significantly expanded the capabilities of SERS for pesticide detection under practical conditions [39]. Therefore, the construction of high-performance, application-oriented SERS platforms necessitates a holistic consideration of both the physicochemical properties of target molecules and the structural and electronic attributes of substrate materials [40].

In summary, surface-enhanced Raman spectroscopy (SERS) has gained recognition as a powerful analytical technique for the trace-level detection of organophosphorus pesticide (OPP) residues, owing to its high sensitivity, molecular specificity, and adaptability to complex sample matrices. However, its broader implementation remains limited by challenges such as substrate batch variability, quantitative instability under matrix interference, and insufficient robustness in field applications. While several recent reviews have documented advances in SERS for pesticide detection, particularly in substrate design, enhancement mechanisms, and spectral interpretation, these studies have generally lacked a dedicated focus on OPPs. Specifically, they often overlook how the molecular structures of OPPs influence SERS signal behavior. To fill this gap, the present review offers a mechanism-driven synthesis of recent developments in SERS-based OPP detection, with particular emphasis on structure–activity relationships, key functional group interactions, and dominant enhancement pathways. In addition, it critically evaluates persistent analytical bottlenecks, including reproducibility under matrix complexity [41,42]. Distinct from previous broad-spectrum reviews, this work also highlights emerging strategies that integrate machine learning, quantum chemical modeling, and rational substrate engineering to enhance analytical performance. Together, these insights provide a strategic framework for advancing the standardization and practical application of SERS in pesticide residue monitoring within food safety and environmental surveillance systems [43].

## 2. Fundamental Principles of SERS and Its Advantages in Molecular Detection

SERS is a highly sensitive molecular detection technique that significantly amplifies Raman scattering signals by inducing localized enhancement effects at the surfaces of noble metal nanostructures. By combining the unique molecular fingerprint recognition capabilities of Raman spectroscopy with the signal amplification benefits derived from localized surface plasmon resonance (LSPR), SERS enables the efficient detection of target species at ultra-low concentrations [44]. This technique is particularly suitable for the rapid identification of trace substances within complex matrix environments. In recent years, propelled by rapid advancements in nanoscience and interfacial engineering, SERS has evolved into a leading-edge technology for trace molecular analysis. Its practical value has been widely demonstrated in key fields, including food safety, environmental pollutant tracking, and qualitative analysis of biological components [45]. 

**Figure 2:**
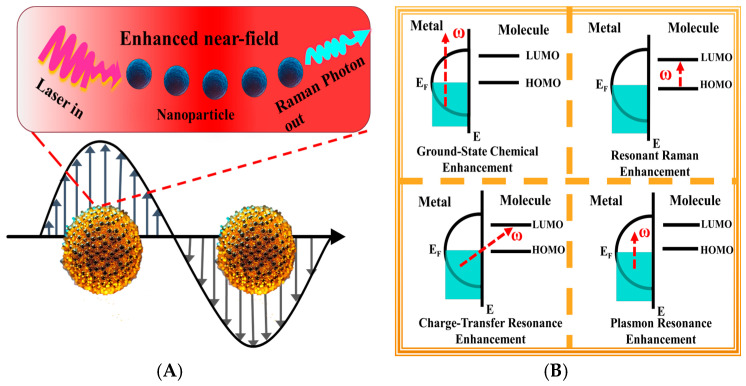
Schematic diagram of SERS mechanism: electromagnetic (EM) enhancement (**A**) Reproduced from [Jian-Feng Li, Yue-Jiao Zhang, Song-Yuan Ding, Rajapandiyan Panneerselvam, and Zhong-Qun Tian, Chemical Reviews, 2017], with permission from [American Chemical Society] [54] and chemical (CE) enhancement (**B**) [55].

The core enhancement mechanisms of SERS can be attributed to the synergistic effects of two physical-chemical processes: electromagnetic enhancement (EM) [46] and chemical enhancement (CE) [47]. EM is the primary source of signal amplification, rooted in the LSPR phenomenon triggered in noble metal nanostructures (Figure 2A) [48]. Upon laser irradiation, the free electrons within the metal undergo resonant oscillation, generating intense localized electric fields, commonly known as hot spots, which are typically formed at sharp edges, nanogaps, and particle junctions. These intense fields can increase the scattering cross-section of nearby target molecules by several orders of magnitude, with the signal theoretically proportional to the fourth power of the local electric field intensity [49]. Through precise control over the morphology, size, spacing, and arrangement of metallic nanostructures, the local electromagnetic field distribution can be effectively tuned, enabling programmable regulation of the SERS signal. Ag and Au nanostructures remain the mainstream choices for SERS substrates due to their excellent plasmonic properties [50,51]. High-curvature nano-stars, multi-branched nanoflowers, and layered core–shell composite structures have been confirmed to generate strong electromagnetic field aggregation at hot spots [52,53].

To address challenges such as signal non-uniformity and batch-to-batch variability, researchers have proposed integrating plasmonic nanostructures with support substrates, including metal–carbon heterostructures, alloyed metal–metal designs, and magnetically responsive composites. These strategies not only improve substrate stability and practical usability but also expand the structural and functional diversity of SERS platforms. While EM achieves substantial signal amplification, CE also plays a crucial role in improving molecular recognition specificity (Figure 2B). CE primarily depends on interactions between target molecules and the metal surface [56,57], including coordination bond formation, electron transfer, and orbital energy level coupling. Upon stable adsorption onto the metal substrate, slight rearrangements in the electronic structure of the molecule can lead to increased Raman activity. Particularly, when the excitation wavelength closely matches the charge transfer transition energy of the molecule–metal complex, additional signal enhancement through photoinduced charge transfer resonance can occur, especially evident in specific systems [58]. It is important to note that both EM and CE are not independent but rather operate as a dynamic, synergistically coupled system [59,60]. The full EM and subsequent optimal signal response are only realized when target molecules are effectively adsorbed at hot spots and establish stable electronic coupling with the metal substrate. Therefore, enhancing SERS performance requires not only the construction of structurally optimized nanostructured substrates but also the implementation of chemical modifications and interfacial engineering to improve molecular adsorption, orientation, and energy level alignment [61,62].

From the perspective of analytical performance, SERS exhibits a range of advantages that are difficult to achieve by traditional techniques. Its most remarkable feature is its extremely high sensitivity: under optimized substrate and detection conditions, SERS can achieve detection limits at the picomolar (pM) level or even lower, enabling direct observation of single-molecule events in carefully controlled systems. However, such performance is seldom realized in complex food matrices due to matrix interference and variability in real-world conditions. Even so, SERS retains the intrinsic vibrational features of target molecules, offering high molecular specificity and spectral identifiability, which makes it particularly suitable for distinguishing analytes in complex samples containing structurally similar interfering substances. Moreover, SERS supports label-free, in situ, and non-destructive detection processes, making it ideal for the analysis of biological samples, food products, and environmental pollutants [63,64]. Its operational procedures are relatively simple, and the detection process is rapid, and in many cases can be performed without large, fixed laboratory instruments. With ongoing miniaturization of Raman hardware, integrated SERS devices that combine fiber-optic probes, microfluidic modules, and portable Raman spectrometers are emerging as candidates for field-oriented, near real-time measurements. However, full on-site deployment remains constrained by practical factors, including laser stability over varying environmental conditions, reproducible and standardized substrate preparation, and adequate control of temperature, humidity, and contamination. Addressing these constraints is essential before routine, fully portable real-time applications can be realized [65].

## 3. Evolution of SERS Substrates and Interface Engineering in OPP Detection

The capability of SERS to achieve highly sensitive detection of trace OPP molecules fundamentally relies on the effective regulation of localized electromagnetic fields within metallic nanostructures [66,67]. Through the rational design of SERS substrates, including careful control of morphology, material composition, and interfacial properties, intense localized enhancement hot spots can be constructed on the metal surface, significantly increasing the Raman scattering cross-section of target molecules [68]. This enhancement enables the previously weak Raman signals to be detected with a high signal-to-noise ratio even in complex backgrounds [69]. As the core component of SERS substrates, nanomaterials critically determine the overall performance of the detection platform in terms of sensitivity, selectivity, stability, and reproducibility [70].

Based on current research advancements, SERS substrate materials can generally be classified into three main categories [71,72]: (i) noble metal nano-component materials (e.g., Au, Ag), (ii) carbon-based and two-dimensional nonmetallic materials (e.g., graphene, carbon nanotubes), and (iii) bimetallic composite structures (e.g., core–shell, alloyed, and heterogeneous structures) [73]. In recent years, emerging liquid metals have been introduced into SERS platform designs, primarily due to their self-reconstructing capabilities and flexible response characteristics. These materials offer novel strategies for expanding detection scenarios and improving substrate stability. In the following sections, the structural optimization strategies, enhancement mechanisms, and performance adaptability of these material systems for constructing SERS substrates in OPP detection were systematically analyzed [74].

### 3.1. Electromagnetic Enhancement Properties of Noble Metal Nanostructures and the Corresponding Geometrical Modulation Strategies

SERS fundamentally depends on the EM effect, which originates from localized surface plasmon resonance (LSPR) effects generated by noble metal nanostructures. Among these, Ag and Au nanoparticles are most commonly employed due to their strong localized plasmonic activity [75,76]. When incident light interacts with these nanostructured surfaces, it induces collective oscillations of conduction electrons, resulting in localized electromagnetic hot spots capable of amplifying the Raman scattering signal of proximate analytes by several orders of magnitude. This enhancement is particularly vital for the detection of trace-level OPPs in complex food matrices, where both sensitivity and molecular specificity are essential. The intrinsic plasmonic properties of noble metals, combined with their tunable morphology and surface chemistry, make them ideal candidates for constructing SERS-active substrates designed for food safety monitoring [77,78,79].

To illustrate the practical application of noble metal nanostructures in SERS, several representative morphologies are noteworthy. Spherical Au nanoparticles are widely used due to their excellent colloidal stability and ease of functionalization; however, their relatively low curvature results in only moderate electromagnetic enhancement. In contrast, Ag nano-cubes and nano-octahedrons offer superior enhancement efficiency owing to their sharper edges and higher plasmonic activity but are prone to oxidation and chemical degradation, limiting their stability in real-world detection environments [80]. Anisotropic structures, such as Au nano-stars and Ag nanoflowers, offer a promising compromise: their high-aspect-ratio tips and branched morphologies promote the generation of densely distributed hot spots with elevated signal intensity. Nevertheless, challenges such as synthetic complexity, morphological instability, and batch-to-batch variability persist, posing barriers to large-scale production and reproducible pesticide analysis [81].

Continued advancement in noble metal-based SERS substrate design requires not only precise morphological engineering but also a deep understanding of the analyte–substrate interaction mechanisms. For OPP molecules containing functional groups such as P=O and P=S, which exhibit strong coordination affinity toward metal surfaces, maximizing the exposure of specific crystallographic facets (e.g., {111} in Au and {100} in Ag) and controlling interparticle spacing are critical for enhancing selective adsorption and hotspot generation. The incorporation of ordered nanoparticle assemblies and three-dimensional nanostructured frameworks has shown further promise in improving hotspot density and spatial uniformity of signal distribution [82,83]. Ultimately, the advancement of robust, reproducible, and high-sensitivity noble metal-based scattering SERS platforms for food analysis is contingent upon the optimization of enhancement efficiency, substrate stability, and scalable fabrication techniques that are amenable to routine application within complex sample matrices [84].

### 3.2. Synergistic Role of Carbon-Based Substrates with Metallic Nanostructures in Hybrid SERS Platforms

Although carbon-based 2D materials are classified as nonmetallic substrates in our taxonomy, they are frequently integrated with plasmonic metals in practical SERS applications. This section therefore focuses on elucidating their synergistic roles within such hybrid SERS systems. Two-dimensional (2D) carbon-based materials have emerged as indispensable components in the design of multifunctional SERS substrates due to their distinctive physicochemical properties and strong synergistic interactions with noble metal nanostructures [85]. Materials such as graphene, graphene oxide (GO), and carbon nanotubes (CNTs) exhibit high surface area-to-volume ratios, tunable surface chemistries, and exceptional electron transport capabilities. These features collectively enhance molecular adsorption, facilitate efficient charge transfer, and contribute to improved hotspot distribution [86]. While noble metals primarily contribute to EM, carbon-based materials support CE mechanisms via π–π stacking, hydrogen bonding, and electrostatic interactions with analyte molecules [87,88]. When combined with plasmonic metals, these hybrid systems provide dual-mode enhancement, offering increased sensitivity, molecular selectivity, and structural stability for the SERS-based detection of trace-level OPPs in complex food matrices.

Several representative examples underscore both the advantages and limitations of carbon-based hybrid SERS platforms [89]. Graphene, with its fully conjugated π–electron system, facilitates strong π–π interactions with aromatic pesticide molecules, thereby prolonging surface residence time and enhancing Raman signal intensity [90]. However, pristine graphene is inherently hydrophobic and chemically inert, which can hinder the dispersion and surface functionalization in aqueous systems [91]. In contrast, GO is abundant in oxygen-containing functional groups, such as carboxyl and hydroxyl moieties, which facilitate electrostatic and hydrogen-bonding interactions with highly polar OPPs, including malathion and phoxim. While the enhanced hydrophilicity improves dispersibility and substrate uniformity, over-functionalization may disrupt π-conjugation and diminish its electrical conductivity. CNTs, characterized by their high aspect ratios and hollow tubular structures, provide extended interfacial contact areas with metal nanoparticles and target molecules [92,93]. They also offer excellent electron transport pathways and mechanical resilience; however, their integration into SERS systems often necessitates complex surface modification protocols and precise control over structural alignment, which may complicate large-scale fabrication [94].

Beyond their individual functionalities, carbon-based materials address several critical challenges in practical SERS applications, including hotspot heterogeneity, substrate instability, and signal irreproducibility in complex sample environments. By tuning analyte–substrate interactions at the nanoscale, these materials enhance molecular recognition efficiency and contribute to more consistent signal generation. Recent studies have demonstrated the successful deployment of graphene–metal and CNT–metal hybrid substrates for the on-site detection of OPP residues in fruits, vegetables, and agricultural water sources, achieving high signal-to-noise ratios even under high-background interference [95]. Furthermore, the mechanical flexibility and thermal durability of carbon-based composites indicate their potential suitability for developing wearable, portable, or field-deployable SERS platforms. At present, however, applications in OPP detection remain largely at the proof-of-concept or laboratory stage, and systematic field trials and robustness tests are still required to demonstrate their practical reliability. Future research should therefore prioritize the rational engineering of carbon–metal interfaces, refinement of surface functionalization techniques, and development of scalable, cost-effective manufacturing processes to advance these materials toward real-world food safety surveillance [96,97].

### 3.3. Interfacial and Electronic Coupling in Bimetallic Structures

Bimetallic nanostructures have attracted considerable attention in the development of advanced SERS substrates, offering an effective strategy to address the inherent limitations of monometallic noble metals, including limited chemical stability, restricted plasmonic tunability, and suboptimal surface interactions [98]. By integrating two metallic elements, most commonly Ag and Au, bimetallic systems exhibit synergistic effects that enhance both electromagnetic EM and CE mechanisms [99,100]. Among these, core–shell and alloyed configurations are the most extensively studied. Core–shell architectures allow for precise modulation of LSPR, control of surface electronic density, and improved resistance to environmental degradation. At the metal–metal interface, differences in carrier density promote interfacial charge redistribution, thereby amplifying local electromagnetic fields and facilitating hotspot formation. In parallel, alloyed bimetallic systems enable atomic-level mixing of two or more metals, enhancing the spatial overlap of enhancement mechanisms, optimizing energy-level alignment, and improving molecular adsorption. These interfacial and electronic advantages make bimetallic nanostructures particularly well-suited for the sensitive and stable SERS-based detection of OPPs in complex food matrices.

Several representative bimetallic nanostructures highlight the functional versatility and technical challenges of these platforms. Ag@Au core–shell nanoparticles, for example, combine the strong plasmonic performance of Ag with the oxidative stability of a thin Au shell. This configuration maintains high signal intensity while improving chemical durability, making it suitable for aqueous food systems. However, excessive Au thickness can attenuate the local field, thereby reducing enhancement [101,102]. Conversely, Au@Ag core–shell structures reverse this arrangement, placing stable Au at the core and highly plasmonic Ag on the surface. While these exhibit stronger SERS signals, they are more prone to oxidation, limiting their long-term stability. Ag@Pd nanostructures introduce a palladium shell that enhances surface adsorption and selectivity, particularly for polar OPPs. Nonetheless, the inherently weaker plasmonic properties of Pd can dampen signal intensity if the shell is too thick. More complex architectures, such as Au@Ag@Au multilayer structures with a Ag interlayer encapsulated between Au shells, demonstrate excellent dual-mode enhancement and improved chemical resistance, although their multi-step synthesis presents challenges for large-scale production [103].

Beyond core–shell architectures, alloyed Au-Ag nanoparticles offer an alternative strategy by embedding both metals within a single crystalline lattice via co-reduction techniques. These structures exhibit tunable optical properties, uniform distribution of enhancement sites, and improved structural robustness [104,105]. For instance, Au-Ag alloy nanoparticles have been successfully employed for the detection of OPPs such as dichlorvos and phoxim, demonstrating enhanced signal uniformity and reproducibility compared to their monometallic counterparts. However, maintaining strict control over atomic ratios and alloy homogeneity is essential, as slight compositional deviations can significantly impact LSPR behavior and surface reactivity [106,107].

In summary, bimetallic nanostructures, whether in core–shell or alloyed form, play a critical role in addressing key challenges in food-related SERS applications, including substrate stability, signal consistency, and compatibility with complex sample matrices [108]. Their ability to engineer interfacial electronic interactions supports the development of highly responsive, chemically stable sensing platforms. Future research should prioritize scalable and environmentally sustainable synthesis approaches, integration with molecular recognition elements for improved specificity, and theoretical modeling to guide rational interfacial design. With continued advancement, bimetallic substrates are poised to become foundational components in next-generation SERS sensors for the rapid, sensitive, and reliable detection of pesticide residues in food safety monitoring [109].

### 3.4. Emerging Material Platforms for Enhanced SERS Detection of Organophosphorus Pesticides

With the rapid advancement of materials science, a variety of novel materials, such as liquid metals, quantum materials, metasurfaces, and room-temperature ambient-pressure (RTAP) superconductors, have emerged, offering new opportunities for enhancing surface-enhanced Raman scattering (SERS) performance in the detection of organophosphorus pesticides (OPPs). These materials exhibit distinct physical and chemical properties, including tunable localized surface plasmon resonance (LSPR), optimized charge-transfer pathways, enhanced electromagnetic hotspots, and excellent biocompatibility, enabling superior sensitivity, selectivity, and real-time detection capabilities.

Liquid metal nanodroplets, particularly those based on gallium, have gained attention as tunable plasmonic materials due to their fluidic nature, high free electron density, and strong LSPR activity [110,111]. When miniaturized to the nanoscale, liquid metal droplets exhibit collective oscillations of free electrons similar to noble metal nanoparticles, consistent with the Drude model [112,113]. Their LSPR properties are tunable with particle size, and reported enhancement factors can reach 10^5^–10^6^ under controlled experimental conditions, approaching those of some Au and Ag nanostructures. However, such performance is highly dependent on substrate design and measurement setup, and has not yet been systematically validated for OPP detection in complex food matrices. Gallium nanodroplets may retain plasmonic activity despite the presence of a thin Ga_2_O_3_ surface oxide layer (approximately 1–3 nm), whereas thicker Ag_2_O layers are known to attenuate Ag signals, but the actual advantage of oxide layers remains context-specific and requires further evidence. Properties such as relative oxidation resistance and nanoscale spacing control have been demonstrated in proof-of-concept studies, but their contribution to OPP SERS performance has yet to be established [114,115]. In terms of biocompatibility, gallium-based droplets have shown high cell viability in certain in vitro studies (greater than 95%), while Ag nanoparticles have been reported to induce cytotoxic effects in some models; nevertheless, these outcomes are assay-dependent and influenced by particle chemistry, dose, and exposure conditions [116]. These features make liquid metal platforms highly suitable for SERS detection of OPPs in complex food matrices and even within biological environments.

Quantum materials offer a complementary enhancement mechanism through quantum confinement, energy band modulation, and interfacial charge-transfer tuning [117]. Atomically thin metal nanosheets and semiconductor quantum dots (e.g., MoO_3_·xH_2_O) can generate strong electromagnetic hotspots and enable multiresonant charge-transfer pathways with analyte molecules, significantly boosting chemical enhancement [118,119]. Additionally, quantum materials support hot-electron injection, coupled resonance effects, and optomechanical spring interactions that further improve signal stability and amplification. These materials may be promising for applications that demand high sensitivity and improved biocompatibility; any extension to in vivo pesticide tracking should be considered exploratory and would require rigorous assessment of toxicological profiles, biodistribution and clearance, delivery routes and dosing, as well as compliance with ethical and regulatory requirements.

Metasurfaces, composed of subwavelength artificial structures, allow precise control of light–matter interactions and have shown significant promise in SERS enhancement [120]. Metallic and dielectric metasurface elements can localize strong electromagnetic fields through LSPR or bound states in the continuum (BIC), creating high-density hotspots. Hybrid metasurfaces, such as Au@MoS_2_, have been reported to simultaneously optimize the laser excitation wavelength (λ_laser_), molecular resonance (λ_mol_), and charge-transfer resonance (λ_CT_), thereby forming so-called triple-resonance platforms. While such systems demonstrate enhanced sensitivity and selectivity in proof-of-concept studies, their specific performance for OPP detection in food matrices remains limited, and they are therefore best considered as a promising direction for future exploration. Moreover, tunable metasurfaces based on graphene–metal heterostructures can dynamically match resonance conditions via voltage control, enabling adaptive SERS platforms tailored to specific analytes [121].

RTAP superconductors are an emerging topic with theoretical interest for SERS but limited relevance to near-term OPP sensing in food matrices. Superconducting states can, in principle, influence electromagnetic field localization and magnetic control of nanostructures; however, experimental validation for OPP detection is scarce, and practical integration with plasmonic architectures remains at the concept stage [122,123]. Although superconductivity has been reported in atomically thin noble metals under specific conditions, evidence linking such systems to robust OPP SERS performance is currently insufficient. Potential hybrids, for example Ag-coated high-Tc superconductors, should therefore be viewed as prospective research directions pending data on hotspot stability, thermal management, and analytical performance in realistic food matrices [124]. While challenges remain regarding material compatibility, temperature–pressure constraints, and surface structure optimization, this direction opens promising avenues for ultra-sensitive, real-time pesticide residue detection [125].

**Figure 3:**
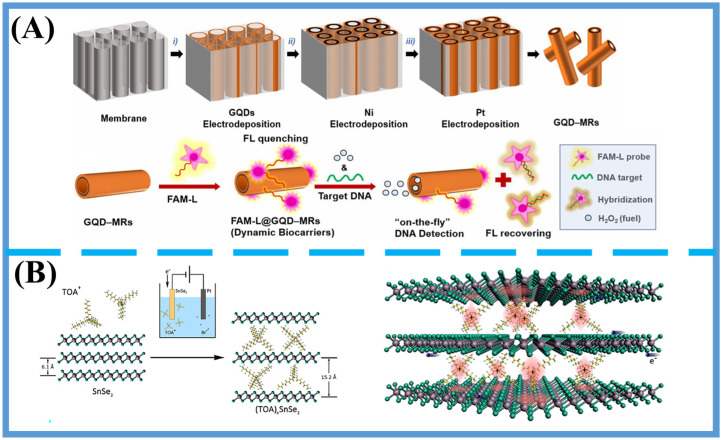
Representative schematics of emerging SERS-active substrates. (**A**) Self-propelled graphene quantum dot microrobots (GQD–MRs) biofunctionalized with DNA probes for dynamic, fluorescence-based “on-the-fly” biosensing with high sensitivity [126]. (**B**) Organic–inorganic hybrid superlattices engineered via electrochemical intercalation of bulky organic cations into layered crystals, introducing charge disorder and quasi-two-dimensional superconductivity, thereby enabling the emergence of quantum Griffiths singularity (QGS) in bulk superconductors [127]. Reproduced from [Yingcheng Zhao, Yueqi Su, Yuqiao Guo, Jing Peng, Jiyin Zhao, Chenyang Wang, Linjun Wang, Changzheng Wu, and Yi Xie, ACS Materials Letters, 2021] [127], with permission from [American Chemical Society].

In summary, these emerging material systems, liquid metals, quantum materials, metasurfaces, and RTAP superconductors offer unique advantages in electromagnetic field manipulation, molecular selectivity, and substrate adaptability. Their integration into SERS sensing platforms holds strong potential to advance the detection of trace OPP residues in food safety and environmental applications. Representative examples of emerging SERS-active substrates are shown in Figure 3. Self-propelled graphene quantum dot microrobots (GQD–MRs), biofunctionalized with DNA probes, integrate the intrinsic features of graphene quantum dots—including water solubility, fluorescent activity, and π–π stacking interactions—with the autonomous mobility of microrobots, thereby enabling real-time “on-the-fly” DNA detection with high sensitivity and selectivity [126]. In parallel, organic–inorganic hybrid superlattices fabricated by intercalating bulky organic cations into layered inorganic crystals introduce charge disorder and quasi-two-dimensional superconductivity, leading to the emergence of quantum Griffiths singularity (QGS) in bulk superconductors. Beyond advancing the understanding of quantum phase transitions in disordered systems, these hybrid superlattices also hold promise as unconventional SERS-active platforms due to their unique charge-transfer properties, enhanced spin–orbit coupling, and structural robustness [127]. Table 1 summarizes the performance characteristics of commonly studied SERS substrate categories, including noble metal nanostructures, carbon-based and two-dimensional nonmetallic materials, bimetallic composites, and emerging materials in the context of organophosphorus pesticide (OPP) residue detection. Key metrics such as synthesis cost and difficulty, signal repeatability, enhancement advantages, and practical limitations are evaluated. The aim is to highlight the trade-offs between sensitivity, stability, scalability, and integration feasibility across different substrate platforms, providing practical guidance for substrate selection and optimization in both laboratory and field-deployable SERS systems.

## 4. Summary of SERS Applications in the Detection of OPPs

SERS has been widely utilized for the detection of OPPs due to its exceptional sensitivity and molecular specificity. In practical applications, the intensity and reliability of the SERS response are largely governed by the chemical structure of the analyte and its interaction with the substrate surface [128]. Key factors influencing signal output include the type and spatial distribution of functional groups, the three-dimensional conformation of the molecule, and its adsorption behavior on plasmonic surfaces. OPP molecules typically contain a combination of functional groups that contribute to SERS activity, such as P=O, (P=S), aromatic rings, halogen substituents (e.g., C-Cl), and polar heteroatoms like nitrogen and oxygen. Among these, the P=O and P=S groups are the most actively involved in surface interactions through coordination bonding and electron donation. For instance, the P=S group in malathion exhibits a strong Raman signal in the 1330–1350 cm^−1^ range, particularly when adsorbed on Ag-based substrates. Similarly, the P=O group in triazophos contributes prominently through stable complex formation at the metal surface [129].

Aromatic rings also play a secondary, yet significant, role by participating in π–π interactions with metallic surfaces, particularly Au. These interactions enhance signal stability and often generate well-defined skeletal vibrational peaks in the 970–1050 cm^−1^ range. Halogen atoms, such as chlorine in dichlorvos or chlorpyrifos, can influence the Raman response indirectly by altering intramolecular electron distribution, resulting in observable bending modes between 600 and 800 cm^−1^. The spatial conformation and rigidity of the molecule further influence its adsorption geometry and orientation within SERS hotspots. Rigid and planar molecules, such as triazophos and phoxim, tend to produce stronger and more reproducible signals due to favorable surface alignment. In contrast, flexible molecules with rotatable bonds may display weaker or shifted signals as a result of conformation-dependent adsorption and diminished surface contact

Moreover, molecules containing multiple SERS-active functional sites often exhibit synergistic signal enhancement and more complex spectral profiles. For example, methyl parathion, which possesses both P=S and aromatic moieties, shows dual-site resonance effects, generating intense and well-resolved peaks. This synergistic behavior facilitates the simultaneous identification of multiple residues within complex sample matrices. In summary, the structural characteristics of OPP molecules, particularly the nature, distribution, and spatial orientation of functional groups, play a critical role in modulating their SERS response. Understanding these structure–response relationships provides a theoretical foundation for accurate spectral interpretation and supports the rational design of SERS substrates tailored to specific pesticide analytes.

### 4.1. SERS Detection and Vibrational Mode Analysis of Representative OPPs

Following the elucidation of the mechanisms by which the molecular structure of organophosphorus pesticides (OPPs) influences their SERS responses, it is essential to investigate the actual spectral behavior of representative OPP molecules under experimental conditions. This includes a comprehensive analysis of their enhancement performance, spectral features, and mechanistic variations across different SERS detection platforms. OPPs differ in their molecular conformation, functional group composition, and spatial orientation, resulting in distinct adsorption behaviors on plasmonic metal surfaces. These variations affect their charge transfer capabilities and interactions with localized electromagnetic fields, thereby leading to notable differences in Raman signal intensity, enhancement factors, and characteristic peak positions.

**Figure 4:**
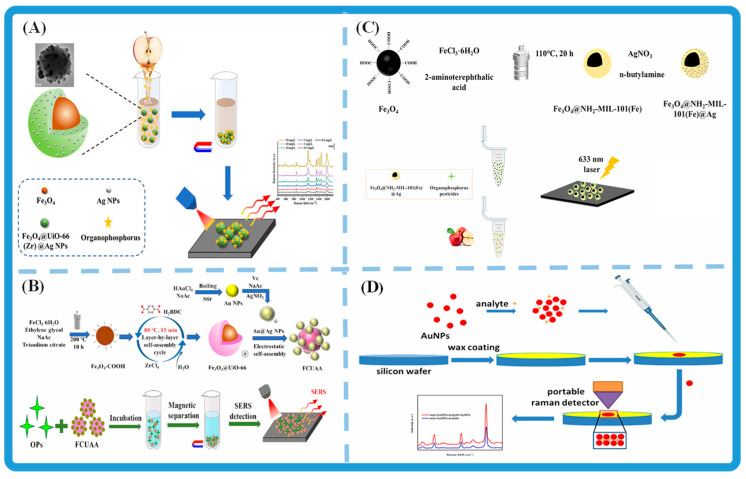
Schematic illustration of Fe_3_O_4_@UiO-66(Zr)@Ag nanoparticles prepared as SERS substrates for the sensitive detection of OPPs [130] (**A**). Preparation process of the FCUAA SERS substrate and its application in the detection of OPP standards [131] (**B**). Preparation process and sensing mechanism of the FNMA SERS substrate for OPP detection [132] (**C**). Schematic diagram of a SERS platform with a AuNPs double-decker structure on a wax-coated surface for trace detection [133] (**D**).

Figure 4 illustrates representative SERS platforms developed for the detection of organophosphorus pesticides (OPPs). Core–shell–satellite Fe_3_O_4_@UiO-66(Zr)@Ag nanoparticles have been fabricated to achieve highly sensitive detection of OPP residues [130]. A dual core–shell magnetic substrate (FCUAA), prepared by assembling Au@Ag nanoparticles onto Fe_3_O_4_@UiO-66, combines strong enrichment ability with magnetic separability, enabling rapid and selective analysis of OPP standards [131]. Similarly, a magnetic nanosensor (FNMA) based on aminated Fe-based MOFs decorated with Ag nanoparticles enhances analyte adsorption and electromagnetic coupling, thereby allowing ultrasensitive detection of OPPs in complex matrices such as apple juice [132]. Furthermore, a wax-coated SERS platform employing a double-decker AuNPs configuration improves analyte confinement and signal reproducibility, facilitating portable, trace-level detection with a handheld Raman detector [133].

**Figure 5:**
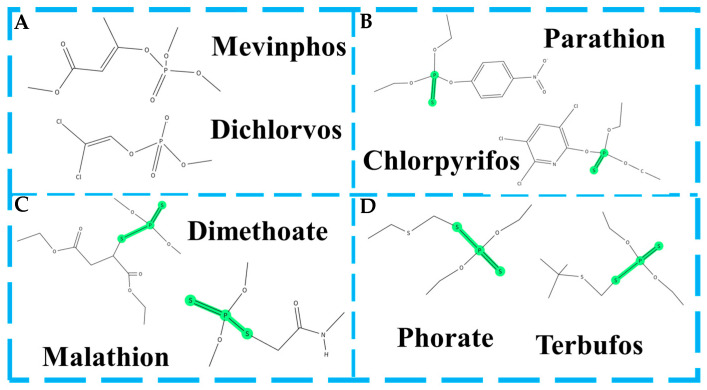
Structural Classification of Organophosphorus Pesticides (OPPs) Based on Sulfur Bonding Configurations. OPPs with zero (**A**), one (**B**), two (**C**) and three (**D**) sulphur atoms.

Among the various molecular determinants of SERS activity, the bonding configuration surrounding the phosphorus atom, particularly the number and position of sulfur atoms, plays a central role. Based on this criterion, OPPs can be broadly classified into four structural categories, each exhibiting distinct SERS behaviors. Non-thionophosphates, such as Mevinphos and Dichlorvos, contain phosphoryl (P=O) groups without sulfur atoms, typically resulting in weak metal surface adsorption and low Raman signal intensities. In contrast, monothiophosphates, including Parathion and Chlorpyrifos, possess a thiophosphoryl (P=S) bond that facilitates stronger coordination with noble metal substrates. Dithiophosphates, such as Malathion and Dimethoate, incorporate both P=S and P–S–R bonds, offering multiple adsorption sites and enhanced signal responses. Polythiophosphates, exemplified by Phorate and Terbufos, feature three or more sulfur atoms in varied bonding configurations, often resulting in complex adsorption dynamics and the appearance of multi-band vibrational features. This structural classification, illustrated in Figure 5, provides a mechanistic framework to underpin SERS performance in OPP detection. Building on this foundation, the following sections systematically examine five representative OPPs: triazophos, phoxim, dichlorvos, methyl parathion, and malathion by comparing their enhancement mechanisms, characteristic vibrational modes, and interactions with SERS substrates. These analyses aim to construct a molecular-level structure–response correlation method to inform the rational research of highly sensitive and selective SERS platforms for multi-residue pesticide detection in food systems. In the following section, five commonly studied organophosphorus pesticides (OPPs) frequently reported in the existing literature will be analyzed in detail. These selected compounds are among those previously discussed in this review.

#### 4.1.1. Triazophos

Triazophos is an organophosphorus insecticide widely used in the control of agricultural crop pests [134]. Its molecular structure contains a triazole ring, an aromatic ring, and a phosphonyl group, which collectively confer strong biological activity and broad-spectrum insecticidal efficacy. However, its high toxicity and persistence also render its monitoring in food and environmental samples particularly critical. From a molecular structure perspective, the Raman activity of triazophos primarily arises from the P=O bond, C-N bond, and aromatic ring framework. These functional groups exhibit pronounced signal enhancement when interacting with metallic nanomaterials. In SERS detection, the characteristic stretching vibration of the P=O bond typically appears at 1270 cm^−1^. This peak is significantly enhanced due to the strong coordination interaction between the lone pair electrons of the phosphonyl group and the surface atoms of the metal, producing a notable CE effect. Additionally, the C-N bond vibration around 1000 cm^−1^ and the aromatic ring skeletal breathing mode near 977 cm^−1^ tend to form stable adsorption through π–electron conjugation interactions with the metal nanoparticle surface. This facilitates uniform molecular distribution on the SERS substrate and improves signal reproducibility. The presence of the aromatic ring in the triazophos molecule provides inherent π–π interaction sites, enhancing its Raman activity on Ag and similar metal surfaces, thereby significantly increasing detection sensitivity.

Experimental studies utilizing Fe_3_O_4_@MIL-100(Fe)@Ag nanocomposite SERS substrates have demonstrated excellent performance in the detection of triazophos [135,136]. These hybrid nanomaterials combine the strong plasmonic resonance of Ag nanoparticles with the magnetic separation capability of Fe_3_O_4_ cores, facilitating efficient enrichment and sensitive detection of target analytes. Under optimized conditions, the system achieved a detection limit (LOD) of 0.038 μM and an enhancement factor (EF) of 2.02 × 10^4^. The method also exhibited a favorable linear response and high reproducibility. Moreover, successful application in real samples, such as apples, further confirmed its practical value for pesticide residue analysis in agricultural products. In another example, Au@Ag core–shell nanoparticles assembled on flexible substrates enabled label-free and portable detection of triazophos on fruit surfaces, achieving an LOD of 0.0032 μM with spike recoveries ranging from 93.36% to 123.6%, thus demonstrating suitability for on-site monitoring [137]. Additionally, a UiO-66@TiO_2_/Au nanohybrid was developed for the rapid SERS detection of triazophos in apple juice. The inclusion of TiO_2_ enhanced the photocatalytic stability of the substrate and improved spectral reproducibility under matrix interference. Collectively, these studies highlight the versatility and efficacy of nanostructured and composite SERS substrates for trace-level detection of triazophos across diverse food matrices, underscoring their potential for practical deployment in food safety monitoring.

It is important to note that the SERS response of triazophos is not only attributed to the electromagnetic enhancement induced by the metallic nanostructure, but also benefits from the synergistic contributions of multiple vibrational modes within the molecular structure. In particular, structurally optimized Ag nanoparticles exhibit significantly enhanced local field intensities at their edges and vertices, forming multiple hotspots that further amplify the Raman signal of adsorbed molecules. Furthermore, the triazole ring in triazophos may, under specific conditions, form stable adsorption complexes with the metal surface, potentially undergoing partial charge redistribution during electron transfer processes, thereby modulating the Raman scattering intensity. In short, triazophos exhibits distinct vibrational responses in SERS detection systems, with its enhancement mechanism jointly governed by chemical coordination, π–π interactions, and localized electromagnetic fields. These features provide well-defined signal characteristics and a robust basis for molecular identification. With ongoing advancements in SERS substrate material design, combined with magnetic separation techniques and portable detection platforms, the efficient, rapid, and quantitative detection of triazophos is becoming increasingly feasible, particularly in applications related to food safety monitoring and environmental contamination traceability.

#### 4.1.2. Methyl Parathion

Methyl parathion is a highly toxic OPP widely employed for the control of pests and diseases in cereal crops, fruits, vegetables, and various economic plants. Its molecular structure consists of an aromatic ring, a P=S group, and an ester group, with the molecular formula C_8_H_10_NO_5_PS [138]. These structural features endow it with high lipophilicity and biological activity, while also posing significant risks of environmental persistence and food contamination, necessitating the development of highly sensitive detection technologies. Its SERS activity primarily originates from three key functional groups: the P=S bond (Raman peak around 1335 cm^−1^), the C–O bond (1150 cm^−1^), and the aromatic ring C–H bending vibration (840 cm^−1^). The P=S group, due to its lone pair electrons, readily forms stable coordination complexes with metal surfaces, facilitating electron transfer during adsorption and significantly enhancing its Raman activity. Meanwhile, the C–O bond and aromatic ring engage in multiple interactions with the Ag nanoparticle surface, including electromagnetic enhancement and π–π stacking effects, which further contribute to signal amplification.

SERS substrates constructed using Ag nanoparticles, combined with sensitive signal acquisition systems, enable efficient recognition of methyl parathion in complex samples. Experimental results have demonstrated that within the concentration range of 0.076–76 μM, this method exhibits a strong linear response (R^2^ = 0.953–0.976), with a detection limit as low as 0.00410 μM [139]. In another study, a GO/ZrO_2_ hybrid SERS substrate was developed for the detection of methyl parathion, where the high surface area and electron transfer capability of GO enhanced signal intensity, while ZrO_2_ provided selective affinity through acid-base interactions. This platform achieved a detection limit of 0.12 μM, a linear range up to 10 μM, and demonstrated good reproducibility in spiked samples with intra-day RSDs < 4.5% and spike recoveries of 97.4–102.1% in matrices such as strawberries, black tea, and irrigation runoff water [140]. Additionally, a flexible AuNP paper-based SERS sensor was fabricated for rapid on-site analysis. Owing to its high hotspot density and substrate portability, the device reached a detection limit of 0.011 μg/cm^2^ for methyl parathion residues on fruit surfaces, with a linear range of 0.018–0.354 μg/cm^2^ and recovery rates ranging from 94.09% to 98.72% in spiked apple samples. These examples highlight the adaptability of methyl parathion to different substrate architectures and the importance of substrate design in enhancing sensitivity, selectivity, and practical applicability across various food systems [141].

From the perspective of the enhancement mechanism, the Raman signal intensification of methyl parathion is determined by the synergistic effect of CE from the P=S group and electromagnetic enhancement from the aromatic ring. The electron distribution within the molecule facilitates electronic migration and local field reconfiguration upon adsorption to the metal surface, thereby significantly amplifying the target signal. As a result, methyl parathion exhibits favorable SERS responsiveness, particularly under conditions where the noble metal substrate is well-designed and surface uniformity is optimized. This enables trace-level quantitative analysis and provides important support for ensuring the safety of fruit and vegetable products and promoting green, sustainable development in agricultural production.

#### 4.1.3. Phoxim

Phoxim is a moderately toxic OPP commonly used to control soil-dwelling pests in crops such as fruit trees, vegetables, and cotton. It exhibits strong stomach and contact toxicity. With the molecular formula C_12_H_15_NO_3_PS, phoxim features several Raman-active functional groups, including a phosphonyl group (P=O), a C-N bond, and an aromatic ring [142]. These structural features impart strong adsorption affinity and electronic coupling capabilities on metallic nanoparticle surfaces, making phoxim an ideal target molecule for SERS detection. In the SERS spectrum, the P=O stretching vibration at 1275 cm^−1^ represents the most prominent Raman signal of phoxim. This enhancement arises from the strong polarizability and coordination behavior of the P=O bond, which forms a stable surface complex with the metal, thereby amplifying the Raman response. Additionally, the C-N stretching vibration (1010 cm^−1^) and the aromatic ring skeletal breathing mode (970 cm^−1^) also show enhanced signals, which are further strengthened by π–π stacking interactions between the molecular π-system and the metal surface.

In experimental studies, a ternary Fe_3_O_4_@UiO-66(Zr)@Ag composite nanomaterial was employed as an SERS-active substrate [130]. This platform integrates the localized surface plasmon resonance (LSPR) effect of Ag nanoparticles, a high-density distribution of electromagnetic hotspots, and the magnetic enrichment capability of the Fe_3_O_4_ core. Under optimized conditions, the system enabled ultrasensitive detection of phoxim, achieving a detection limit (LOD) of 0.144 μM, an enhancement factor (EF) of 5.62 × 10^6^, and a linear range of 0.352–175.87 μM, while maintaining high signal stability and reproducibility in complex matrices such as apple juice. In a separate study, a flexible SERS substrate consisting of Ag nanoparticles embedded in a PDMS film demonstrated high sensitivity and reusability for phoxim detection on fruit and vegetable surfaces, achieving an LOD of 0.0552 μM with a linear range of 0.0352–17.587 μM and recovery rates ranging from 91.7% to 105.6%, confirming its potential for field application. Additionally, a GO/Au NP cellulose membrane substrate was constructed, where the mixed cellulose ester membrane supported layer-assembled GO to promote phoxim adsorption and Au NPs to facilitate strong SERS signal enhancement. This configuration enabled stable phoxim detection in chrysanthemum samples, underscoring the substrate’s adaptability for phoxim and the key role of substrate structure in enhancing SERS detection performance in herbal plants [143].

Mechanistically, the P=O group enhances the Raman signal through chemical adsorption on the Ag surface, accompanied by localized electron density redistribution. Meanwhile, the C-N and aromatic structures, due to their structural rigidity and π-conjugation ability, contribute to the overall polarizability, further intensifying the SERS response. Thus, phoxim’s enhancement is governed by a synergistic effect of chemical adsorption and electromagnetic field amplification, ensuring high detection sensitivity and reliability. In summary, phoxim exhibits well-defined Raman peaks and strong signal intensity under SERS conditions. The clear understanding of its interaction mechanisms with metallic nanostructures makes it an important model compound for evaluating SERS platform sensitivity. This technique holds great promise for practical applications in pesticide residue monitoring on fruits and vegetables as well as in environmental sample screening.

#### 4.1.4. Dichlorvos (DDVP)

Dichlorvos is a highly toxic and volatile organophosphorus insecticide widely used in grain storage, pest control, and agricultural pest management. Its molecular formula is C_4_H_7_Cl_2_O_4_P, featuring a phosphonyl group (P=O) and two chlorinated functional groups (C-Cl), making it a molecule of strong polarity [144]. Due to its poor chemical stability and propensity for degradation, the rapid detection of dichlorvos using SERS techniques has attracted considerable attention. In the SERS spectrum, dichlorvos exhibits two main enhanced vibrational modes: a P=O stretching vibration at 1265 cm^−1^ and a C-Cl bending mode at 745 cm^−1^. The former shows strong Raman activity, resulting from coordination between the lone pair electrons on the P=O group and metal surface atoms, which induces significant CE. Although the intrinsic Raman activity of the C-Cl group is relatively weak, it can be markedly amplified under local electromagnetic field enhancement conditions.

In a separate study, Au@Pt core–shell nanoparticles were incorporated into an enzyme inhibition-based SERS sensor, employing acetylcholinesterase (AChE) as a biorecognition element. This system exhibited high selectivity for dichlorvos through specific enzymatic inhibition, achieving a detection limit of 0.091 μM in pear samples. Additionally, a rippled silicon substrate modified with Ag nanoparticles (AgNPs) was developed for label-free SERS detection, achieving an enhancement factor of approximately 10^7^ and enabling detection of dichlorvos at 4.53 μM without the need for chemical pretreatment. These examples underscore the versatility of SERS-based dichlorvos detection across various substrate configurations. The integration of nanostructure design with biochemical recognition and surface engineering strategies significantly enhances analytical performance and supports practical deployment in food safety and environmental monitoring [145,146].

Within the dichlorvos molecule, the C-Cl group exhibits mild affinity for noble metal surfaces, such as Ag and Au, through weak physisorption. In contrast, the P=O group primarily governs the stability of adsorption and facilitates electron transfer at the substrate interface. This dual-group coordination mechanism is essential for the molecule’s SERS responsiveness and serves as a distinctive feature for differentiating dichlorvos from other organophosphorus compounds. In conclusion, dichlorvos presents highly sensitive vibrational fingerprints in SERS detection systems, enabling rapid response to ultra-trace levels of the analyte. The technique offers strong technical support for fast, non-destructive detection of highly toxic pesticides in environmental and food samples and is particularly suited for residue screening during distribution and emergency monitoring scenarios.

#### 4.1.5. Malathion

Malathion is a low-toxicity organophosphorus insecticide widely used in the protection of fruits, vegetables, horticultural crops, and for public health pest control [147]. Its molecular formula is C_10_H_19_O_6_PS_2_, and its structure comprises a P=S group, two ester groups (C–O), and other sulfur-containing functional groups, making it a typical multifunctional organophosphorus compound. While malathion exhibits relatively good biodegradability, its extensive use in agricultural systems raises concerns regarding residue accumulation, underscoring the importance of developing rapid and sensitive detection techniques. In SERS detection, malathion displays multiple characteristic enhancement peaks, with the most prominent at 1330 cm^−1^, corresponding to the P=S stretching vibration. This signal is significantly enhanced due to the strong coordination ability of the lone pair electrons on the P=S bond, enabling robust chemical adsorption onto the metal surface. The C–O bond contributes another key peak, typically observed around 1145 cm^−1^, where electrostatic or hydrogen-bonding interactions between its polar groups and the metal substrate help stabilize the adsorption state of the molecule, thereby reinforcing the Raman response.

In a recent study, Shu et al. developed a multifunctional SERS nanoprobe (MOFTb@Au@MIP) for the highly sensitive and selective detection of malathion [148]. The platform integrates a terbium-based metal–organic framework (MOFTb) with molecularly imprinted polymers (MIP) and Au nanoparticles, enabling recognition and catalytic amplification simultaneously. Using this approach, malathion was detected with a limit of detection (LOD) as low as 0.182 nM, based on a linear calibration model spanning from 4.54 nM to 63.6 nM (R^2^ = 0.971). The method exhibited excellent reproducibility, with intra- and inter-day relative standard deviations (RSDs) below 8%, and achieved recovery rates between 95.2 and 107.4% in spiked fruit juice samples. Matrix interference tests confirmed high specificity even in the presence of structurally analogous pesticides. Mechanistically, the P=S group serves as the primary chemically adsorbed site, forming stable coordination complexes with noble metal surfaces, which enhances the molecule’s electronic polarizability and Raman scattering cross-section. The MIP layer further improves selectivity through shape-specific recognition, while the MOF structure enhances enrichment capacity. Together, these interactions result in a highly efficient and stable signal enhancement system. In summary, malathion exhibits clear advantages in SERS detection due to its multiple active vibrational modes, well-defined characteristic peaks, and high affinity for metal substrates. Supported by rationally designed nanocomposite substrates, this approach shows significant practical potential for detecting residues in food and monitoring environmental contamination. Future research may incorporate molecular simulations and data-driven optimization strategies to systematically analyze adsorption orientations, resonance conditions, and spectral behaviors, thereby enabling more precise quantitative detection and large-scale sample screening.

Table 2 summarizes the structural features, SERS-active Raman peaks, substrate compositions, and analytical performance metrics of representative organophosphorus pesticides (OPPs) analyzed using various SERS platforms. Substrates include noble metals, bimetallic structures, MOFs, COFs, and hybrid composites tailored for enhanced sensitivity and specificity. The selected examples cover a range of OPPs categorized by sulfur atom content in their chemical structures, providing a systematic overview of how different molecular features (e.g., P=O, P=S, C-Cl, C-O, C-N bonds) influence Raman signal intensity under distinct substrate configurations. Analytical performance is detailed in terms of linear detection ranges and limits of detection (LODs), with corresponding literature references to ensure traceability. This comparative summary provides a practical reference for researchers developing or selecting SERS substrates for real-world food and environmental monitoring applications involving pesticide residue analysis.

### 4.2. Structural Enhancement Mechanisms and Application Summary of SERS in OPP Detection

This section systematically discusses the enhancement mechanisms and molecular response characteristics of SERS for the detection of representative OPPs, with particular emphasis on how key functional groups within pesticide molecules interact with metal substrates through coordination adsorption, electron transfer, and π–π interactions to significantly amplify Raman scattering signals. On this basis, for pesticide molecules featuring multiple coexisting functional groups (e.g., malathion and methyl parathion), the synergistic enhancement between P=O/P=S groups and aromatic ring structures becomes especially prominent—manifesting as multi-channel enhancement pathways, multi-peak spectral responses, and high signal-to-noise ratio profiles. Furthermore, the molecular conformational rigidity and coplanarity of these functional groups also play a critical role in influencing adsorption adaptability to metal surfaces: molecules with high rigidity, concentrated substitution sites, and coplanar configurations are more likely to form efficient electronic coupling at nanoscale hotspots, thereby further improving overall detection sensitivity and reproducibility. Mechanistically, the entire SERS response process can be described as a multi-stage dynamic synergy that encompasses adsorption localization (driven by functional group-substrate interactions), charge coupling (facilitated by electronic transfer between molecules and substrates), polarizability enhancement (modulated by molecular conformational features), and Raman cross-section amplification induced by the intrinsic structural characteristics of OPP molecules [159].

Based on this understanding, future development of SERS for OPP detection should prioritize the construction of structure–response mapping models, integrating quantum chemical modeling, machine learning algorithms, and spectral analysis to improve detection predictability for new pesticide molecules. At the same time, several bottlenecks need to be addressed before practical implementation. These include batch-to-batch variability in nanoparticle synthesis that affects hotspot reproducibility, matrix adsorption competition in complex food samples that interferes with target recognition, and quantitative signal drift caused by substrate instability and environmental fluctuations. Addressing these challenges will require the development of highly stable, matrix-tolerant, and uniform SERS substrates capable of delivering consistent sensitivity and reproducibility. With such improvements, SERS can more effectively transition from material-focused innovations toward mechanism-informed and platform-integrated applications in agricultural product safety monitoring and environmental surveillance.

## 5. Challenges and Prospects of SERS Technology in OPP Detection

Despite the exceptional sensitivity and molecular specificity of surface-enhanced Raman spectroscopy (SERS) for organophosphorus pesticide (OPP) detection, its practical application in food safety monitoring still faces several critical challenges. The reliability of SERS-based quantitative analysis remains an area for optimization, as non-uniformity of enhancement effects and variability in molecular adsorption orientations can potentially compromise quantification accuracy, especially in complex food matrices. While internal standard calibration and machine learning-based signal processing have been explored to stabilize SERS signals, broader standardization for routine detection workflows would benefit from more concrete, application-focused solutions. To address this, recent studies have explored practical strategies centered on signal normalization and interference mitigation, which may help reduce these limitations: ratiometric or isotope-labeled internal standards could counteract adsorption variability by linking target signal intensity to a stable reference, on-substrate references may enable real-time correction of equipment-induced drift during food sample analysis, and multivariate calibration models have shown potential to mitigate matrix interference by decoding overlapping spectral information—collectively supporting reduced quantification error and improved reproducibility in food-related matrices. Beyond the aforementioned challenges, environmental factors (humidity, temperature fluctuations, ambient contaminants, mechanical disturbances) impacting SERS performance under field conditions have received insufficient attention. These factors significantly undermine signal reproducibility/stability—e.g., high humidity induces Ag-based nanoparticle oxidation, while temperature variations cause spectral drift. Such effects are negligible in controlled labs but become pronounced during on-site analysis. Thus, future field-deployable SERS systems should integrate environmental regulation (humidity control, temperature compensation, substrate encapsulation) plus automated sample delivery and vibration isolation. These additions further elaborate on the challenges of translating SERS from lab demonstrations to practical on-site detection platforms.

In this context, the integration of quantitative structure–activity relationship (QSAR) models offers a promising avenue for refining SERS system design for OPP detection. QSAR models aim to establish correlations between OPP molecular descriptors (e.g., dipole moment, frontier orbital energy gaps) and SERS enhancement performance, which may facilitate two practical applications relevant to food safety testing: predicting the potential detectability of unstudied OPPs based on their structural properties, and guiding the rational design of substrates that might be tailored to specific functional groups (e.g., P=O or P=S) translating theoretical structure response relationships into potentially useful tools for optimizing SERS platforms in food analysis. A recent study illustrates this potential, using QSAR to link molecular properties to enhancement factors and validate preliminary substrate design guidelines.

Continued optimization of substrate design, particularly with functional nanomaterials such as molecularly imprinted polymers (MIPs) and metal–organic frameworks (MOFs), is widely recognized as important for enhancing target specificity and suppressing background interference in food sample analysis [160,161,162]. The potential benefits of these materials lie in their complementary functionalities: MIPs typically incorporate selective recognition cavities that tend to prioritize adsorption of target OPPs over structural analogs, which may improve detection selectivity; MOFs often provide uniform pore structures and analyte enrichment sites that can contribute to standardized analyte–substrate interactions, potentially reducing signal variability (RSD); together, these features may enhance matrix tolerance by minimizing non-target adsorption and signal drift-addressing some key barriers to the practical application of SERS in food safety monitoring. Specific cited studies have begun to quantify these benefits, supporting the utility of MIPs and MOFs beyond theoretical concepts.

## 6. Conclusions and Future Outlook

SERS is increasingly recognized as a powerful analytical technique for the trace-level detection of OPPs, owing to its high sensitivity, molecular specificity, and rapid analytical throughput. Recent research efforts have focused on the development of multifunctional nanostructured substrates, including noble metal nanoparticles, core–shell architectures, bimetallic hybrids, and MOF-based systems that enable dense hotspot formation and tunable surface chemistries to enhance molecular recognition [163,164,165]. Particular emphasis has been placed on leveraging Raman-active functional groups, such as P=O, P=S, and aromatic moieties, to achieve high spectral specificity and signal stability in complex food and environmental matrices.

Emerging directions emphasize the rational design of SERS platforms guided by structure–activity relationships, with computational modeling and data-driven strategies facilitating predictive substrate optimization. The integration of SERS with artificial intelligence (AI) and machine learning (ML) is rapidly advancing, particularly for automated spectral interpretation and high-throughput residue screening. Parallel efforts are focused on the miniaturization of detection systems to enable portable, on-site applications, thereby supporting real-time monitoring throughout agricultural supply chains. The convergence of advanced materials engineering, theoretical simulation, and intelligent data analytics is accelerating the translation of SERS technologies from fundamental research toward practical deployment in food safety and environmental surveillance

The reproducibility of SERS substrates remains a critical bottleneck for their widespread adoption. Recent advancements suggest that industrialization and standardization, combined with substrate batch QC, could be viable solutions to this challenge. Techniques like nanoimprint lithography and roll-to-roll manufacturing enable mass production of uniform SERS substrates with controlled nanostructures. Meanwhile, AI-driven inspection systems can monitor substrate quality in real-time, reducing batch-to-batch variability. Automated Quality Control (AQC) in SERS substrate fabrication integrates machine vision and AI-driven statistical process control (SPC) to achieve real-time monitoring of nanostructure uniformity (e.g., nanoparticle density, plasmonic hotspot distribution), ensuring RSD < 10% in signal enhancement. Advanced systems employ robotic handling to minimize human-induced contamination, while IoT-enabled predictive maintenance optimizes equipment performance (e.g., sputtering targets). Microfluidic SERS chips leverage automated optical inspection (AOI) to detect defects in nanochannel structures, enhancing reproducibility for food safety applications. Additionally, the calibration transfer (CT) can be applied to correct the spectral variations from different substrates. CT is a chemometric method that enables the transfer of analytical models between different SERS substrates to standardize signal responses, addressing variations caused by differences in plasmonic nanostructures, excitation conditions, or instrumentation. Key techniques include direct standardization (DS) for linear intensity corrections, piecewise direct standardization (PDS) for complex spectral adjustments, and slope/bias correction to compensate for systematic deviations. Challenges such as matrix effects and dynamic range limitations require integration with internal standards (e.g., isotopically labeled molecules) and robust normalization methods to ensure accuracy. Moreover, other potential future directions for SERS may include matrix-matched calibration, internal standards, reproducible detection, and external validation, which will be detailed in the Supplementary Materials.

## Figures and Tables

**Table 1:** Comparison of Key Performance Parameters for Representative SERS Substrates.

Classification	Substrate	Cost and Difficulty	Repeatability	Main Advantage	Main Limitations	Ref.
Noble metal nano-component materials	AgNPs	Low and Simple	Moderate (spot-to-spot RSD ≤ ~10–15%)	High plasmonic activity enables ultrasensitive detection with cost-effective synthesis.	Prone to oxidation and batch variability, limiting stability and reproducibility	[75]
	AuNPs	Low and Simple	Good under controlled conditions, but affected by aggregation and sample matrix effects.	High chemical stability, simple synthesis, broad linear range, and good sensitivity	Limited target affinity without modification; performance affected by matrix interference and hotspot uniformity	[77]
Carbon-based and two-dimensional nonmetallic materials	Defect-Engineered MoS	Low material cost, but defect engineering adds complexity	Moderate—Uneven distribution of defects may cause hotspot variability and batch-to-batch inconsistency	Strong chemical enhancement; tunable electronic properties; good for non-metal-interacting targets; biocompatible	Lower SERS enhancement than noble metals; CE-dominant mechanism; sensitive to preparation parameters; limited stability	[88]
	Aligned Carbon Nanotube Arrays	Needs CVD and precise alignment control	Growth uniformity affects results	High surface area; excellent electrical conductivity; flexible integration	Complex fabrication; limited Raman enhancement; poor SERS activity without modification	[89]
	MOF/Graphene Composites	Requires multi-step synthesis and post-treatment	Needs control of MOF loading and dispersion	High surface area, tunable chemistry, synergistic enhancement with graphene	Complex synthesis; stability may vary under different conditions	[90]
	GO–CuInS_2_/ZnS QD Composite	GO and QDs are individually accessible, but integration is multi-step	Depends on QD uniformity and GO dispersion	Broad spectral response; high sensitivity due to QD fluorescence and GO adsorption	Complex interface control; potential quenching or photobleaching effects	[91]
Bimetallic composite structures	Au–Ag Core Shell NPs	Requires controlled seed-mediated growth of Au and Ag shells	Good under optimized prep	Strong synergistic enhancement; high sensitivity and stability in juice matrix	Sensitive to Ag oxidation; requires precise shell thickness control	[102]
	Au@Ag NPs	Requires multi-step synthesis with precise Ag shell growth	Good under controlled conditions; sensitive to shell thickness and oxidation	High enhancement factor, broad-spectrum detection, portable SERS compatibility	Ag shell prone to oxidation; shell thickness control affects performance	[103]
	Ag–Au alloy NPs	Requires precise control over alloy ratio and uniformity	Sensitive to synthesis conditions and aging	Tunable plasmonic properties; better stability than pure Ag	Alloy composition affects reproducibility; costlier than monometallics	[106]
Emerging Material Platforms	Ga-based liquid metal NPs	Requires inert atmosphere, sonication, or microfluidic fabrication methods	Sensitive to oxidation and handling	Excellent biocompatibility, reconfigurability, and self-healing surfaces	Easily oxidized; synthesis not yet scalable; surface control is complex	[111]
	Quantum-cofined nanostructures	High—Requires complex synthesis like MBE, PLD, or ultrafast laser systems	Low/moderate sensitivity to external fields and fabrication defects	Enables tunable many-body interactions and nonlinear optical properties	Limited reproducibility; high sensitivity to environment and defects	[117]
	III–V MQW Dielectric Metasurfaces	Highly requires epitaxial growth and nanofabrication	Precision-dependent	Active tunability, sharp spectral response, CMOS compatibility	High fabrication cost; integration into wet SERS setups is challenging	[120]

**Table 2:** Representative SERS substrates and detection performance for OPPs in food and environmental samples.

Classification of OPPs	Pesticide	Characteristic Raman Peaks (cm^−1^)	Substrate	Characteristics	Analytical Performance	Ref.
No Sulfur Atom	Dichlorvos (DDVP)	P=O (1265) and C-Cl (745)	Au@PtNPs	AChE-mediated SERS sensor enables sensitive dichlorvos detection using Au@PtNPs.	Linear range: 0.02–2 mg/L LOD: 20 μg/L	[145]
	Dichlorvos (DDVP)	P=O (1265) and C-Cl (745)	AgNPs	Rippled Si SERS substrate detects 1 ppm Dichlorvos with ~10^7^ enhancement, no binder needed.	Linear range: 1–100 ppm LOD: 1 ppm	[146]
One Sulfur Atom	Triazophos	P=O (1270) and C-N (1000)	Fe_3_O_4_@MIL-100(Fe)@Ag	Magnetic MOF-based SERS substrate enables rapid, sensitive triazophos detection with high recovery and low LOD.	Linear range: 0.1–50 mg/L LOD: 21 μg/L	[130]
	Triazophos	P=O (1270) and C-N (1000)	Au@AgNPs	Au@AgNPs-based SERS enables rapid, sensitive detection of triazophos and methyl-parathion in peaches.	Linear range: 0.005–10 mg/kg LOD: 0.001 mg/kg	[137]
	Triazophos	P=O (1270) and C-N (1000)	Ag/SB	Ag/SB films enable flexible, stable SERS detection of triazophos with a 2.5 × 10^−8^ M LOD.	Linear range: 0.5–50 μM LOD: 25 nM	[149]
	Methyl Parathion	P=S (1335) and C-O (1150)	GO/ZrO_2_	Hybrid GO/ZrO_2_ SERS substrate enables rapid, sensitive, and label-free detection of methyl parathion via efficient charge transfer.	Linear range: 0.12–10 μM LOD: 0.12 μM	[140]
	Methyl Parathion	P=S (1335) and C-O (1150)	AuNPs	Flexible AuNPs paper SERS detects methyl parathion on-site with 0.011 μg/cm^2^ LOD and high recovery.	Linear range: 0.018–0.354 μg/cm^2^ LOD: 0.011 μg/cm^2^	[141]
	Phoxim	P=O (1275), C-N (1010), and aromatic ring (970)	Fe_3_O_4_@UiO-66(Zr)@Ag nanocomposites	Fe_3_O_4_@UiO-66(Zr)@AgNPs enable sensitive and reliable SERS detection of phoxim.	Linear range: 0.1–50 mg/L LOD: 0.041 mg/L	[136]
	Phoxim	P=O (1275), C-N (1010), and aromatic ring (970)	AgNPs-PDMS	Flexible AgNPs-PDMS SERS enables rapid, accurate detection of multiple pesticides on produce.	Linear range: 50–5000 μg/L LOD: 15.69 μgL^−1^	[150]
	Thiamethoxam	C-N (739) and N-O (866)	UiO-66@TiO_2_	UiO-66@TiO_2_/Au enables rapid SERS detection of thiamethoxam and triazophos in apple juice	Linear range: 10–10,000 μg LOD: 3.51 μg/L	[151]
	Methamidophos	C-S (675) and P-N (938)	Fe_3_O_4_@Ag@COF	Fe_3_O_4_@Ag@COF-based ratiometric SERS sensor enables sensitive, reproducible methamidophos detection in complex matrices, including green tea	Linear range: 0.001–100 μg/L LOD: 8.3 × 10^−5^ mg/kg	[152]
	Chlorpyrifos	P=S (567) and C-Cl (634)	AuNP/HNT	The AuNP/HNT paper substrate enables ultrasensitive, reproducible, and stable detection of chlorpyrifos in agricultural products.	Linear range: 0.01–10 μM LOD: 7.9 nM	[153]
	Chlorpyrifos	P=S (567) and C-Cl (634)	AuNPs-NF	Popcorn-like AuNPs enable portable SERS detection of chlorpyrifos in fruit with 1 μM sensitivity	Linear range: 1.5–6.25μM LOD: 1 μM	[154]
	Chlorpyrifos	P=S (567) and C-Cl (634)	Au@AgNPS	SERS with chemometrics enables rapid, accurate detection of chlorpyrifos residues in tea	Linear range: 3 × 10^−9^–10^−4^ M (Data mining)	[155]
Two Sulfur Atoms	Malathion	P=S (1330) and C-O (1145)	MOF_Tb_@Au@MIP	MOFTb@Au@MIP enables sensitive trimode detection of malathion via SERS, RRS, and Abs signals.	Linear range: 4.54–63.6 nM LOD: 0.182 nM	[148]
	Malathion	P=S (1330) and C-O (1145)	AgNPs	Ag NPs-based SERS enables sensitive, rapid malathion detection in tea with low LOD and high precision	Linear range: 0.1–5 ppm LOD: 0.05 ppm	[156]
	Malathion	P=S (1330) and C-O (1145)	AC@Ag	AC@AgNPs substrate enables rapid, sensitive malathion detection in wheat with strong signals and high model accuracy.	Linear range: 0.1–10 μg/mL LOD: 0.95 mg/L	[129]
	Malathion	P=S (1330) and C-O (1145)	AgNPs	Cl^−^-enhanced AgNPs-SERS detects malathion at 3 ppb with high fruit sample recovery.	Linear range: 5–2000 ppb LOD: 3 ppb	[157]
	Fenthion	P=O (1150) and Aromatic ring (1600)	FCUAA	FCUAA substrate enables rapid, sensitive SERS detection of trace OPs in vegetables.	Linear range: 0.02–10 mg/kg LOD: 12.1 ng/kg	[131]
	Omethoate	P-S (413)	AuNRs	Gold nanoparticle-enhanced SERS enables rapid, quantitative detection and distribution mapping of omethoate and chlorpyrifos residues on apple surfaces.	Linear range: 1.64–8.43 μg cm^−2^ LOD: 1.63 μg cm^−2^	[158]

## Data Availability

The datasets generated during the current study are available from the corresponding author on reasonable request.

## Supplementary Materials

The following supporting information can be downloaded at: https://www.mdpi.com/article/10.3390/foods14213683/s1, File S1: Recent advances in SERS-based for detecting organophosphorus pesticides in food: A critical and comprehensive review.
