# Supplementary material for: Recent Advances in SERS-Based Detection of Organophosphorus Pesticides in Food: A Critical and Comprehensive Review
Source: Foods. 2025 Oct 29;14(21):3683. doi: 10.3390/foods14213683 (PMC12610478; doi:10.3390/foods14213683)
Supplement: Supplementary file 1 [file foods-14-03683-s001.zip › foods-3871491-supplementary.pdf]

Supplementary information

Recent advances in SERS-based for detecting organophospho-rus pesticides in food: A critical and comprehensive review

Kaiyi Zheng[1], Xianwen Shang[1], Zhou Qin[1], Yang Zhang[1], Jiyong Shi[1], Xiaobo Zou[1*] and Meng Zhang[2]

1 School of Food and Biological Engineering, Jiangsu University, Zhenjiang 212013, China;

kaiyizheng@ujs.edu.cn (K.Z.); SXW18906320352@163.com (X. S.); 19599960979@163.com (Z. Q.); yang-zhang1@ujs.edu.cn (Y.Z.); shi_jiyong@ujs.edu.cn (J.S.);

2 Department of Physics, East China University of Science and Technology, Shanghai 200237, China;

* Correspondence: zou_xiaobo@ujs.edu.cn (X.Z.)

Future Directions for SERS Analysis:

(1) Matrix-Matched Calibration

Addressing matrix effects by developing substrate-specific calibration models tailored to complex biological fluids (e.g., serum, saliva) will enhance quantitative accuracy.

Integration of artificial intelligence for dynamic matrix correction could automate calibration workflows in point-of-care diagnostics.

Standardized protocols for matrix selection (e.g., synthetic vs. native matrices) are needed to ensure reproducibility across labs.

(2) Internal Standards

Isotopically labeled molecules or plasmonic nanoparticles with known enhancement factors will serve as robust references for signal normalization.

Dual-functional internal standards (e.g., Raman-active tags with SERS reporters) could simultaneously correct for analyte loss and substrate variability.

Optimization of internal standard concentrations to match the dynamic range of target analytes remains a critical challenge.

(3) Replicated Detection

Statistical validation through multi-site replicated experiments ($\geqslant 3$ independent substrates per analyte) will strengthen confidence in SERS measurements.

Machine learning models trained on large-scale replicated datasets could predict inter-substrate variability and minimize false positives.

Open-access repositories of replicated SERS spectra would accelerate method standardization.

(4) External Validation

Cross-platform validation against reference techniques (e.g., HPLC-MS, ICP-MS) is essential for clinical and environmental SERS applications.

Collaborative round-robin testing involving international labs would establish consensus performance metrics.

(5) Single-Molecule SERS

Ultra-sensitive detection of single molecules requires precise control of hotspot uniformity and minimization of photobleaching.

Advanced plasmonic nanostructures (e.g., DNA origami-enhanced substrates) could enable reproducible single-molecule studies.

New data analysis algorithms are needed to distinguish true single-molecule signals from stochastic background noise.

(6) In Vivo Real-Time Monitoring

Biocompatible SERS probes with long-term stability will facilitate continuous in vivo metabolite tracking.

Minimally invasive fiber-optic SERS sensors could enable real-time monitoring of therapeutic drug concentrations.

Overcoming biofouling through anti-fouling coatings remains a key challenge for clinical translation.

(7) Multimodal Integration

Combining SERS with other techniques (e.g., fluorescence microscopy, mass spectrometry) will provide complementary molecular information.

Hybrid platforms could enable simultaneous structural and compositional analysis of complex samples.

Developing unified data fusion algorithms is critical for extracting comprehensive insights from multimodal datasets.

(8) Machine Learning-Driven SERS

Deep learning models trained on large SERS spectral libraries could accelerate pattern recognition and peak assignment.

Generative adversarial networks (GANs) might help synthesize realistic SERS spectra for training purposes.

Explainable AI methods will be essential to ensure transparency in model-driven SERS analysis.

(9) Green SERS Methods

Developing environmentally friendly plasmonic materials (e.g., copper-based alternatives to gold/silver) will reduce costs and toxicity.

Water-based synthesis protocols could minimize hazardous waste generation in substrate fabrication.

Energy-efficient SERS systems powered by low-power lasers or LED sources may

expand field applications.

(10) Standardization Initiatives

Establishing international consortia to harmonize SERS protocols, reporting formats, and performance metrics.

Developing certified reference materials with well-characterized SERS properties for method validation.

Creating guidelines for SERS-based clinical diagnostics to facilitate regulatory approval pathways.
